# Detection and volume estimation of artificial hematomas in the subcutaneous fatty tissue: comparison of different MR sequences at 3.0 T

**DOI:** 10.1007/s12024-017-9847-8

**Published:** 2017-03-01

**Authors:** Kathrin Ogris, Andreas Petrovic, Sylvia Scheicher, Hanna Sprenger, Martin Urschler, Eva Maria Hassler, Kathrin Yen, Eva Scheurer

**Affiliations:** 1 Institute of Forensic Medicine, Medical University of Graz, Universitaetsplatz 4/II, A-8010 Graz, Austria; 2 Ludwig Boltzmann Institute for Clinical-Forensic Imaging, Graz, Austria; 3 Institute for Biomedical Engineering, Graz University of Technology, Graz, Austria; 4 BioTechMed-Graz, Graz, Austria; 5 Institute for Computer Graphics and Vision, Graz University of Technology, Graz, Austria; 6 Division of Neuroradiology, Vascular and Interventional Radiology, Department of Radiology, Medical University of Graz, Graz, Austria; 7 Institute of Forensic and Traffic Medicine, University of Heidelberg, Heidelberg, Germany; 8 Institute of Forensic Medicine, Health Department Basel, University Basel, Basel, Switzerland

**Keywords:** 3.0 T MRI, Subcutaneous fatty tissue, Hematoma, Porcine tissue model, Volume measurement, Forensic medicine

## Abstract

In legal medicine, reliable localization and analysis of hematomas in subcutaneous fatty tissue is required for forensic reconstruction. Due to the absence of ionizing radiation, magnetic resonance imaging (MRI) is particularly suited to examining living persons with forensically relevant injuries. However, there is limited experience regarding MRI signal properties of hemorrhage in soft tissue. The aim of this study was to evaluate MR sequences with respect to their ability to show high contrast between hematomas and subcutaneous fatty tissue as well as to reliably determine the volume of artificial hematomas. Porcine tissue models were prepared by injecting blood into the subcutaneous fatty tissue to create artificial hematomas. MR images were acquired at 3T and four blinded observers conducted manual segmentation of the hematomas. To assess segmentability, the agreement of measured volume with the known volume of injected blood was statistically analyzed. A physically motivated normalization taking into account partial volume effect was applied to the data to ensure comparable results among differently sized hematomas. The inversion recovery sequence exhibited the best segmentability rate, whereas the T1T2w turbo spin echo sequence showed the most accurate results regarding volume estimation. Both sequences led to reproducible volume estimations. This study demonstrates that MRI is a promising forensic tool to assess and visualize even very small amounts of blood in soft tissue. The presented results enable the improvement of protocols for detection and volume determination of hemorrhage in forensically relevant cases and also provide fundamental knowledge for future in-vivo examinations.

**Electronic supplementary material:**

The online version of this article (doi:10.1007/s12024-017-9847-8) contains supplementary material, which is available to authorized users.

## Introduction

Imaging techniques like computed tomography (CT) and magnetic resonance imaging (MRI) are well established in many clinical applications, and various qualitative findings and quantitative measurements derived from imaging data are used to aid radiologists and clinicians in the diagnosis and follow-up of different diseases. MRI and CT play a major role in tumor diagnosis and staging e.g. in lung cancer [1–3], breast tumors [4–6], prostate tumors [7–10], and brain tumors [11–15], as well as in the follow-up of tumor progression [16, 17], often requiring volume measurements of tumor structures. Another specific parameter is the volume measurement of liquids in the assessment of hemorrhages. Further, volume determination has proven especially useful for measurement of liquid volumes in the assessment of hemorrhages [18], and in the diagnosis and prognosis of cerebral bleedings [19]. In the case of cerebral hemorrhage, determination of both initial hematoma volume and volume change, is essential as both factors are independent predictors of treatment outcome and mortality [20, 21]. Hence, CT and MRI protocols are well established for the determination of these critical factors [19, 22–24]. In contrast, only limited knowledge exists on the detection, volume estimation and investigation of hemorrhage in soft tissue using MRI [25], as such lesions have less therapeutic consequences.

Differently to clinical applications, in forensic medicine information on blunt force injuries, especially hematomas in the subcutaneous fatty tissue, are highly relevant [26]. Analysis of hematomas is required for forensic reconstruction and verification of a specific course of events, especially in cases involving child maltreatment or interpersonal violence. To date, the gold standard for the detection of subcutaneous hematomas due to blunt force is a detailed examination of the entire body surface including photo documentation of any visible lesions [27]. Nevertheless, subcutaneous hematomas are not necessarily visible externally, since visibility depends on many factors, such as time since impact, pigmentation of the skin or the amount of blood in the hemorrhage. In order to obtain additional information regarding blunt force associated with soft tissue injuries, the use of imaging methods, such as CT and MRI has been proven to be beneficial [28].

Due to superior contrast achievable in soft tissue and absence of ionizing radiation, MRI holds advantages over CT, particularly if used in clinical forensic cases involving victims of interpersonal violence. Although nowadays a growing interest using MRI in forensic medicine is recognized, to date there is only limited experience regarding the detection and assessment of forensically relevant soft tissue injuries [28–32].

Therefore, the aim of this study was the experimental determination of the detection limits of subcutaneous blood volumes in a porcine tissue model using 3.0 T MRI. Four MR sequences were evaluated regarding their potential for precise estimation of the blood volume of hemorrhages.

## Materials & methods

### Porcine tissue model preparation

Ten pork bellies from the butcher were prepared by injecting freshly drawn venous blood from a healthy human volunteer into the subcutaneous fatty tissue of the pork belly to create artificial hematomas.

**Fig. 1:**
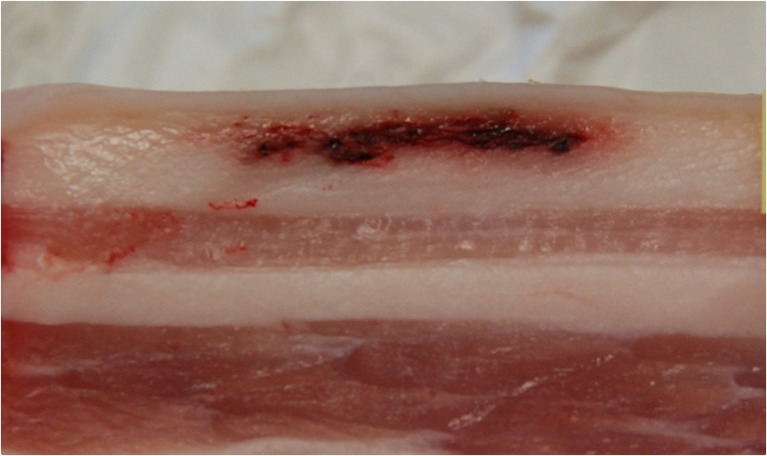
Photograph of a cross section of an artificial hematoma in a pork belly

A total of 40 hematomas were injected into ten pork bellies using four different blood volumes (0.1, 0.3, 0.6 and 1.0 mL). The placement of the hematomas with the four different volumes was performed randomly across the pork bellies. During porcine tissue model preparation care was taken to only introduce hematomas into the subcutaneous fatty tissue layer (Fig. 1). Nevertheless, localization of hematomas in the muscular tissue could not be completely prevented.

### MR imaging

MR images of pork bellies preheated to approximately 37 °C were acquired at 3.0 T (TimTrio, Siemens Healthcare, Erlangen, Germany) using a combination of a spine and small flex coil. The protocol consisted of the following four MR sequences: T1 weighted (T1w) Fast Low Angle Shot (FLASH), T1w Turbo Inversion Recovery (TIR), Proton Density weighted (PDw) Turbo Spin Echo (TSE) with fat saturation (FS), and T1w and T2w TSE sequence (see Table 1). The sequences were chosen in order to maximize contrast between hematomas and surrounding tissues.

**Table 1:** Sequence parameters used for the acquisition of MR images

	FLASH	TIR	PDw TSE FS	T1T2w TSE
Sequence type	FLASH 3D	TIR 2D	TSE 2D	TSE 2D
In-plane resolution (mm^2^)	0.83 × 0.83	0.83 × 0.83	0.72 × 0.72	0.72 × 0.72
Number of slices	40	40	40	40
Slice thickness (mm)	2	2	2	2
TE (ms)	2.02	9	8.8	53
TR (ms)	100	5610	3380	619
TI (ms)	-	200	-	-
Flip angle (°)	44	180/90/180	90/180	90/180
Bandwidth (Hz/Pixel)	620	349	296	296
Averages	1	3	3	3
Fat saturation	-	-	FS	-
Turbo factor	-	7	7	7

*MR* Magnetic Resonance, *TE* Echo Time, *TR* Repetition Time, *TI* Inversion Time, *FLASH* Fast Low Angle Shot, *TIR* T1 weighted Turbo Inversion Recovery, *PDw TSE FS* Proton Density weighted Turbo Spin Echo with fat saturation, *T1wT2w TSE* T1 and T2 weighted TSE sequence

### Image analysis

**Fig. 2:**
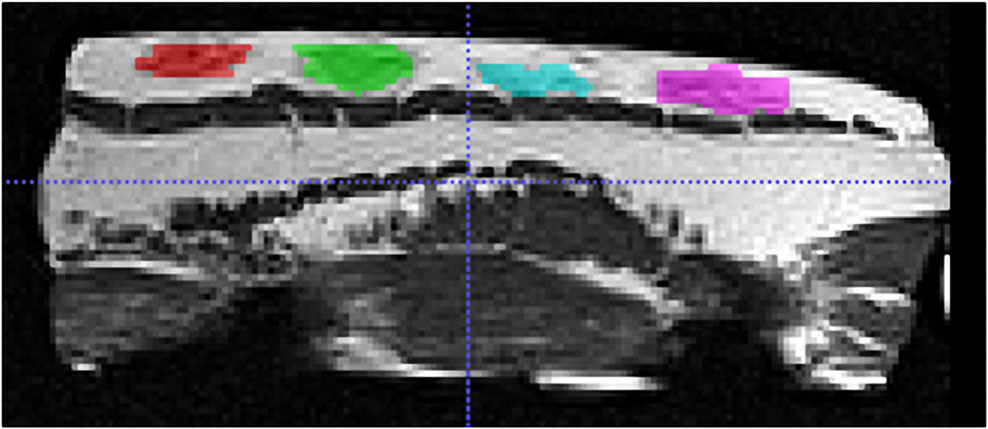
MR (Magnetic Resonance) image of a porcine tissue model acquired with the T1T2w TSE (T1 and T2 weighted Turbo Spin Echo) sequence. *Colors* represent the areas of the four artificial hematomas identified during manual segmentation using ITK-SNAP software

Four blinded observers (3 forensic pathologists, 1 radiologist) conducted manual segmentation of the 160 hematoma scans (ten porcine tissue models, each with four hematomas and four corresponding MR sequences) using the ITK-SNAP [33] software (www.itksnap.org) by selecting all voxels corresponding to the hematoma on each slice of the scan. For image analysis the window level was fixed at Full-Width Half-Maximum for each series and kept the same for all observers. Figure 2 shows an example of a segmented scan.

Hematoma volumes were automatically calculated by the ITK-SNAP software. For assessment of intra-observer variability, segmentation was repeated after one week for 50% of the hematomas (randomly selected).

### Statistical analysis

Frequency cross tabulations were calculated to assess the segmentability of all hematomas, all sequences, and volumes. Hematomas were considered segmentable with a calculated volume larger than 0. An additional forensic pathologist decided whether the hematomas were only located in the subcutaneous fatty tissue or also in the muscular tissue. The location of the artificial lesions was considered in statistical analysis. Accuracy and precision of volume measurements were assessed both quantitatively using summary statistics and graphically using boxplots of the estimated volumes (median, 25th and 75th percentiles, whiskers to demonstrate the lowest/highest point within 1.5 × interquartile range [IQR], data beyond these limits were considered outliers) for each rater, sequence and hematoma size. In all diagrams the estimated volume was plotted against the ground truth volume (injected volume). Analysis was performed twice, initially for all hematomas independent of their location and thereafter for hematomas located in the subcutaneous fatty tissue only. Boxplots show the pooled and averaged results of the forensic pathologists, the radiologist, and finally for all observers.

Intra-observer reliability was determined by Bland-Altman analysis [34], in which the mean value of two ratings was plotted against their difference. Bias and the Limits of Agreement (LoA) were calculated. For the assessment of inter-observer reliability the same analysis was conducted using the differences of the first rating of two distinct observers. For intra- and inter-observer reliability analysis only data from hematomas located in the subcutaneous fatty tissue were taken into account, as the study and the protocol were designed to focus on this issue.

A major requirement for a valid statistical analysis is the homoscedasticity of the differences, i.e. the variance is independent from actual hematoma size. Therefore, the correlation (Kendall’s τ) between mean and absolute value of the differences ($\overset{-}{V}$ vs. |*V*1 − *V*2|) was calculated, to assess if there was a systematic relationship. In case of heteroscedasticity either a log-transform was applied, or the difference was normalized by the mean ($\frac{V1- V2}{\overset{-}{V}}$). Calculation of Kendall’s τ showed that there was significant heteroscedasticity in the differences, i.e. the variance of the differences was dependent on the size of the hematoma. In such cases a suitable transformation of the data was sought. Of all the proposed transformations, a physically motivated transformation model taking the partial volume effect into account removed this heteroscedasticity most effectively. Therefore, this transformation was considered to be the best choice for further analysis and physically motivated normalization was performed: Assuming a hematoma of spherical form with radius ρ and “oversegmenting” a relatively thin layer δ, the resulting error is proportional to ρ^2^δ, or $\sqrt[3]{V^2}$ (Online Resource 1). Hence, the difference was divided by $\sqrt[3]{{\overset{-}{V}}^2}$. After transformation Kendall’s τ was computed once again. Bias and LoA (and their corresponding confidence intervals) were computed for the transformed data according to Bland et al. [34]. Subsequently, those parameters were back-transformed by multiplication with $\sqrt[3]{{\overset{-}{V}}^2}$ to yield curved limits and bias for the original data. For the assessment of intra- and inter-observer variability the data were also transformed prior to the computation of bias and LoA. Subsequently, those values were back transformed and graphically displayed.

## Results

### Visibility/contrast

**Fig. 3:**
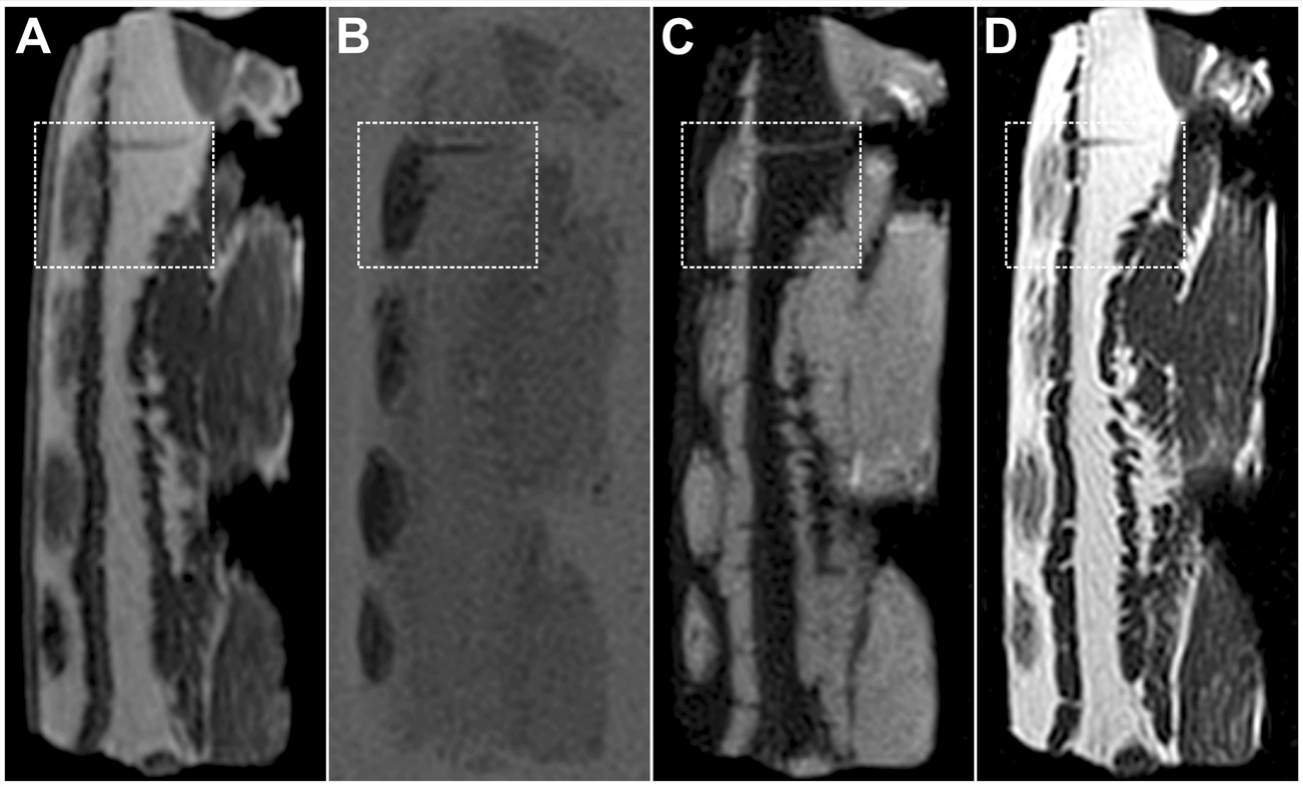
MR (Magnetic Resonance) images of a porcine tissue model using four different MR sequences: **a** FLASH, **b** TIR, **c** PDw TSE FS, **d** T1T2w TSE. The *white rectangles* highlight a hematoma where the blood was partly injected into the muscular layer. (FLASH, Fast Low Angle Shot; TIR, T1 weighted Turbo Inversion Recovery; PDw TSE FS, Proton Density weighted Turbo Spin Echo with fat saturation; T1wT2w TSE, T1 and T2 weighted TSE sequence)

Visual comparison of the four sequences showed good positive or negative contrast between hematomas and the subcutaneous fatty tissue. Hematomas were hyper intense in the PDw TSE FS sequence (Fig. 3c), while in the other sequences (FLASH, TIR, T1T2w TSE) they displayed as hypo intense (Fig. 3a–d). During visual control it was found that 432 out of the 640 segmentations were completely located in the subcutaneous fatty tissue, the remaining 208 were at least partially located in the muscular layers (illustrated by white rectangles in Fig. 3).

### Segmentability

In total, 640 hematomas were assessed using the ITK-SNAP software. 611 out of 640 (95.5%) hematomas were segmentable, with a segmentation rate ranging from 88.8% to 100% depending on the used MR sequence. In contrast to the other sequences, in the TIR sequence all hematomas could be successfully segmented. The segmentation rate concerning injected volumes ranged from 93.8% (0.3 mL) to 96.9% (1.0 mL). Details regarding segmentability of all hematomas are given in Table 2.

**Table 2:** Number of segmented hematomas in relation to the total number of artificially created hematomas, separately for different injected blood volumes and MR sequences

Injected volume	0.1 mL	0.3 mL	0.6 mL	1.0 mL	Total segmentation
MR sequence					
FLASH	40/40	38/40	38/40	36/40	125/160 (95.0%)
TIR	40/40	40/40	40/40	40/40	160/160 (100%)
PDw TSE FS	38/40	40/40	39/40	40/40	157/160 (98.1%)
T1T2w TSE	36/40	32/40	35/40	39/40	142/160 (88.8%)
	154/160 (96.2%)	150/160 (93.8%)	152/160 (95.0%)	155/160 (96.9%)	611/640 (95.5%)

*MR* Magnetic Resonance, *FLASH* Fast Low Angle Shot, *TIR* T1 weighted Turbo Inversion Recovery, *PDw TSE FS* Proton Density weighted Turbo Spin Echo with fat saturation, *T1wT2w TSE* T1 and T2 weighted TSE sequence

When hematomas located only in subcutaneous fatty tissue were separately assessed, 428 out of 432 (99.1%) were segmentable (Table 3), indicating an improved segmentability for all used MR sequences (98.1% to 100%) compared to the segmentation rate when all hematomas were considered.

**Table 3:** Number of segmented hematomas located in the subcutaneous fatty tissue in relation to the total number of artificially created hematomas located in the subcutaneous fatty tissue, separately for different injected blood volumes and MR sequences

Injected volume	0.1 mL	0.3 mL	0.6 mL	1.0 mL	Total segmentation
MR sequence					
FLASH	28/28	26/28	24/24	28/28	106/108 (98.1%)
TIR	28/28	28/28	24/24	28/28	108/108 (100%)
PDw TSE FS	28/28	28/28	24/24	28/28	108/108 (100%)
T1T2w TSE	28/28	26/28	24/24	28/28	106/108 (98.1%)
	112/112 (100%)	108/112 (96.4%)	96/96 (100%)	112/112 (100%)	428/432 (99.1%)

*MR* Magnetic Resonance, *FLASH* Fast Low Angle Shot, *TIR* T1 weighted Turbo Inversion Recovery, *PDw TSE FS* Proton Density weighted Turbo Spin Echo with fat saturation, *T1wT2w TSE* T1 and T2 weighted TSE sequence

TIR and PDw TSE FS sequences allowed segmentation of all hematomas in the subcutaneous fatty tissue. Segmentation rates related to injected blood volumes into fatty tissue ranged between 96.4% for 0.3 mL and 100% for 0.1, 0.6, and 1.0 mL.

### Accuracy and precision

**Fig. 4:**
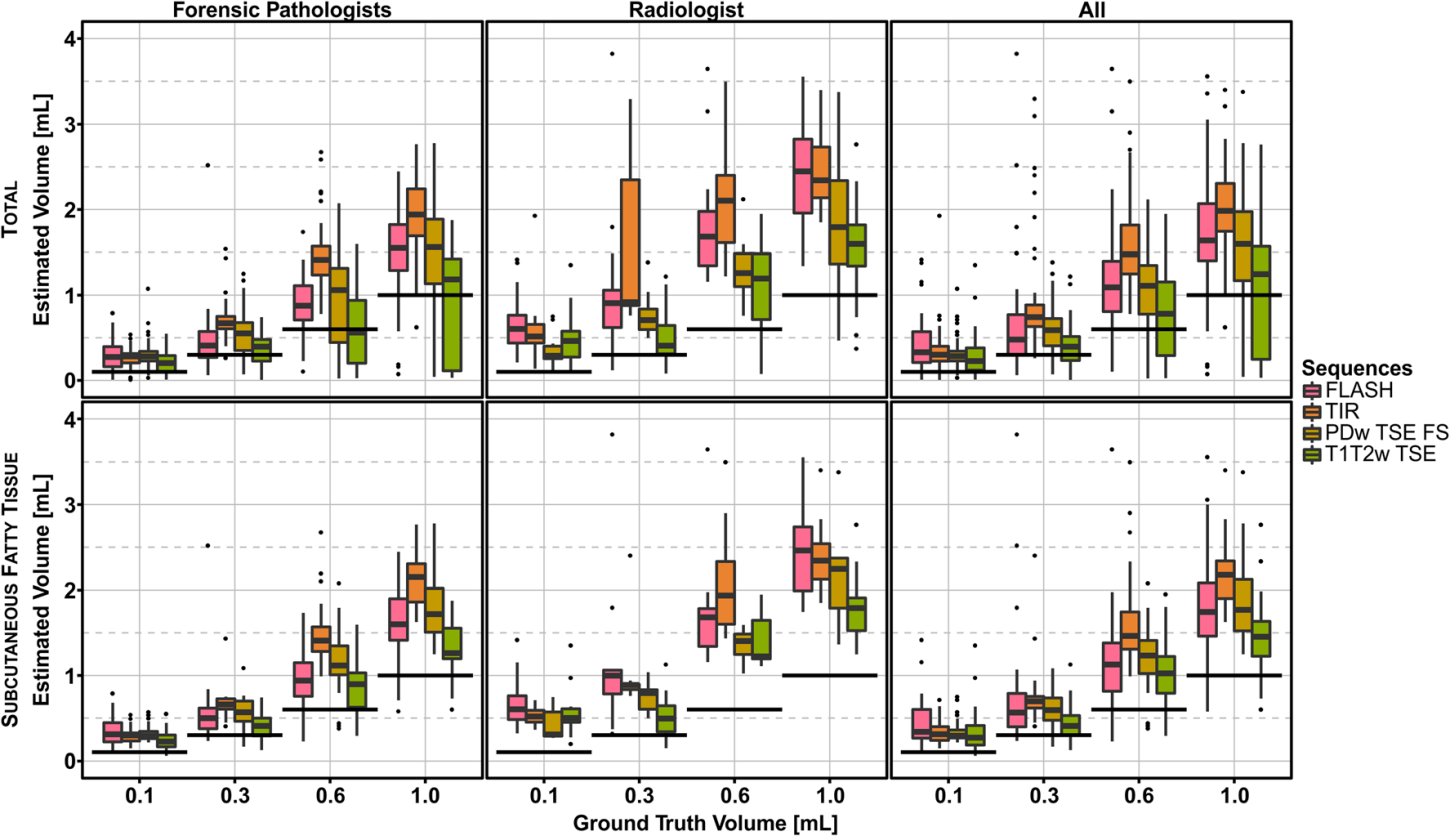
Boxplots of ground truth volume plotted against the estimated hematoma volume. *Top*: Total hematomas, *bottom*: hematomas located only in subcutaneous fatty tissue. *Colors* represent the MR (Magnetic Resonance) sequences used for imaging and further segmentation (in chronological order—*pink*: FLASH, *orange*: TIR, *brown*: PDw TSE FS, *green*: T1T2w TSE). *Boxes* illustrate the median, 25th and 75th percentiles, and whiskers to lowest/highest data point within a 1.5 × interquartile range, *dots* represent outliers. The *thick black lines* demonstrate the ground truth volumes. (FLASH, Fast Low Angle Shot; TIR, T1 weighted Turbo Inversion Recovery; PDw TSE FS, Proton Density weighted Turbo Spin Echo with fat saturation; T1wT2w TSE, T1 and T2 weighted TSE sequence)

The boxplots comparing volume estimation with ground truth (Fig. 4) show that in general the medians of the determined volumes were overestimated compared to the injected volume, independent of the observers, sequences and locations. In comparison to the analysis of all hematomas, those located in subcutaneous fatty tissue showed less outliers and a reduced IQR. This could be particularly well observed in the T1T2w TSE sequence. Comparison of the medians of the estimated volumes of the hematomas located in the subcutaneous fatty tissue showed that these volumes were closest to the ground truth (see Table 4, bold numbers).

**Table 4:** Pooled and averaged results (volumes calculated by segmentation) of all observers: median, 25th (Q 0.25) and 75th (Q 0.75) percentiles of segmented hematomas located in the subcutaneous fatty tissue, bold numbers showing the median closest to the ground truth

Ground truth [mL]	Sequence	Median [mL]	Q 0.25 [mL]	Q 0.75 [mL]
0.1	FLASH	0.34	0.27	0.60
0.1	TIR	0.31	0.24	0.40
0.1	PDw TSE FS	0.29	0.27	0.36
0.1	T1T2w TSE	**0.27**	0.18	0.41
0.3	FLASH	0.57	0.40	0.79
0.3	TIR	0.70	0.62	0.75
0.3	PDw TSE FS	0.60	0.48	0.74
0.3	T1T2w TSE	**0.41**	0.30	0.53
0.6	FLASH	1.13	0.82	1.38
0.6	TIR	1.46	1.31	1.75
0.6	PDw TSE FS	1.23	1.02	1.41
0.6	T1T2w TSE	**1.03**	0.79	1.22
1.0	FLASH	1.75	1.46	2.08
1.0	TIR	2.18	1.90	2.34
1.0	PDw TSE FS	1.82	1.53	2.14
1.0	T1T2w TSE	**1.45**	1.23	1.64

*Q* Quartile, *FLASH* Fast Low Angle Shot, *TIR* T1 weighted Turbo Inversion Recovery, *PDw TSE FS* Proton Density weighted Turbo Spin Echo with fat saturation, *T1wT2w TSE* T1and T2 weighted TSE sequence

### Intra- and inter-observer reliability

**Fig. 5:**
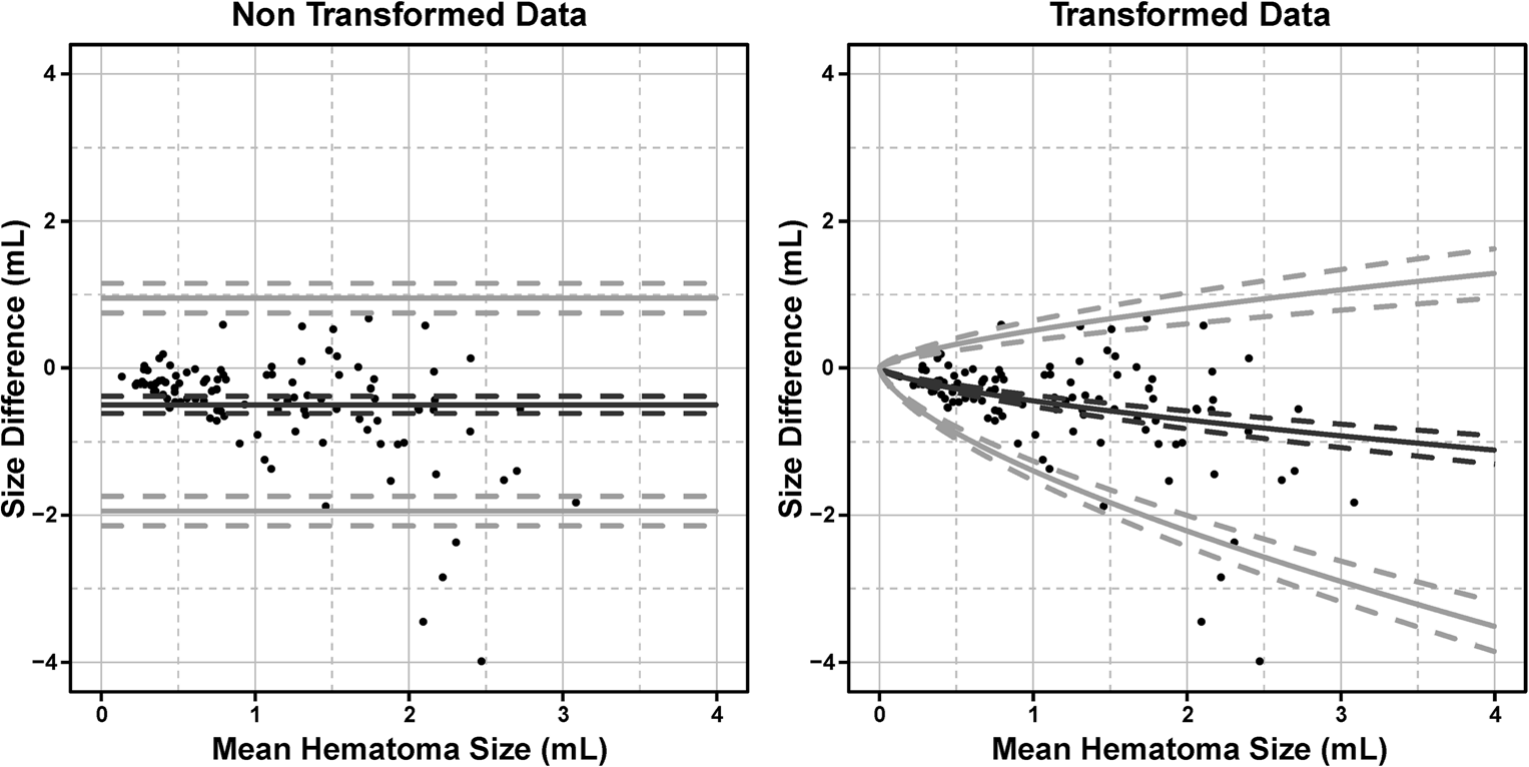
Bland-Altman plot describing inter-observer reliability of one radiologist and one forensic pathologist for non-transformed data (*left*) and transformed data (*right*) of hematomas of the subcutaneous fatty tissue. The mean hematoma size is plotted against the difference in volume estimation. The *black curves* represent the bias, the grey ones the LoA (Limits of Agreement). *Dashed curves* illustrate the 95% confidence interval of bias (*black*) and LoA (*grey*)

Comparison of non-transformed and transformed data using Bland-Altman analysis showed that the LoA tended to be too broad for small hematoma volumes and too narrow for large volumes. This deficiency was effectively removed by applying the physically motivated transformation taking the partial volume effect into account, which yielded curved limits for a better description of the data (Fig. 5).

**Fig. 6:**
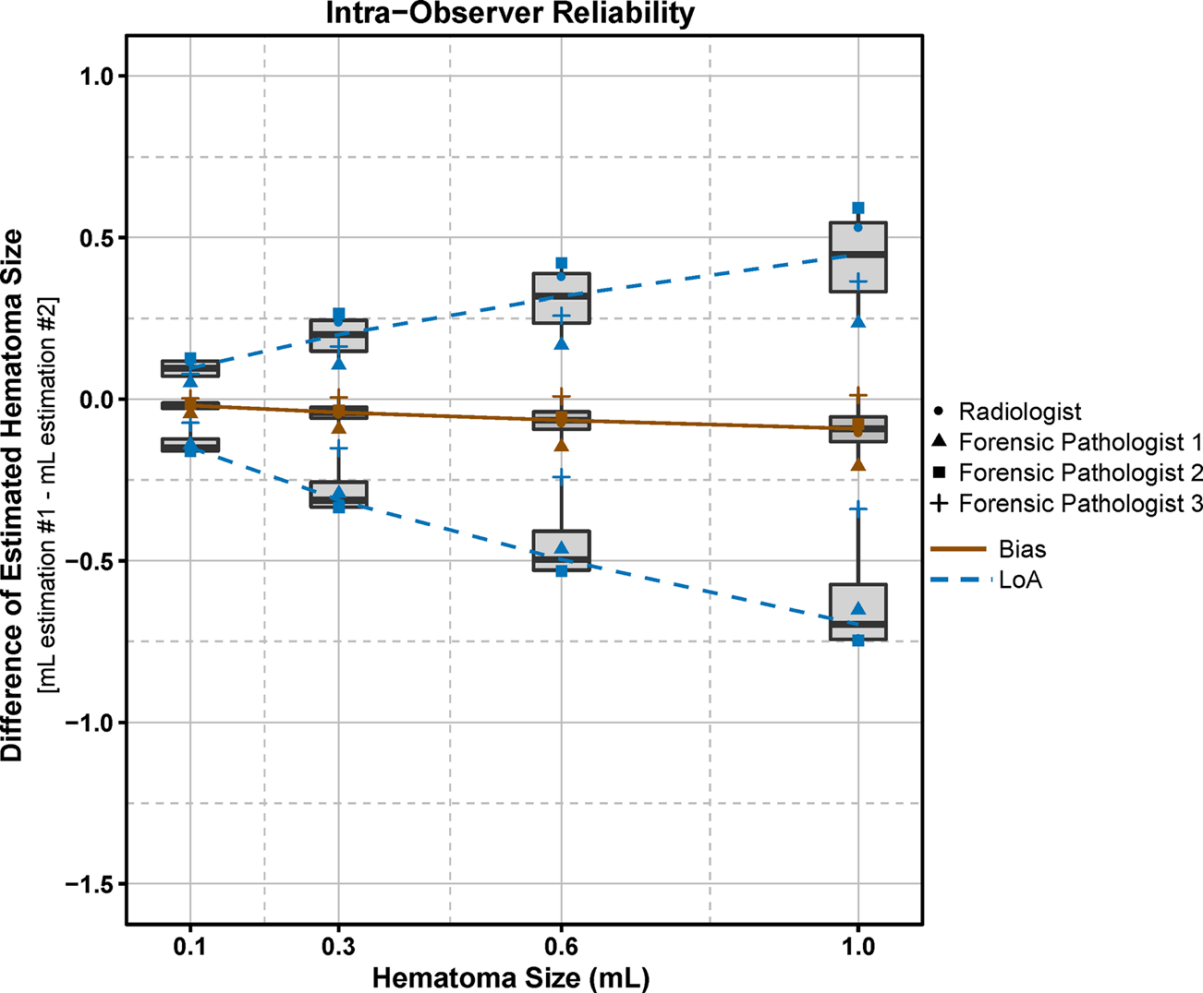
Bland-Altman plot describing intra-observer reliability of summarized results of transformed data (hematomas located in the subcutaneous fatty tissue). *Brown symbols* represent the averaged bias over the estimated hematoma volumes of each observer, out of two measurements. The *blue symbols* represent the LoA (Limits of Agreement; bias ±1.96 × standard deviation) of the estimated hematoma volumes of each observer. The *boxes* represent the median, 25th and 75th percentiles of the averaged data (bias and LoA) over all observers. The *brown continuous curve* links the bias averaged over all four observers (*thick black line* in boxes), the *blue dashed curves* link the averaged LoA of all observers

Figure 6 represents summarized results of intra-observer reliability of estimations of hematoma volumes. On average the slope of the bias curve was negative, i.e. the second volume segmentation tended to be larger than the first. There was substantial variation yielding LoA from −0.15 to 0.10 mL for 0.1 mL hematomas and from −0.70 to 0.45 mL for 1.0 mL. With the TIR sequence, a slight positive bias was observed in contrast to the other sequences. Additionally, the TIR sequences showed lower LoA compared to the other sequences (Online Resource 2).

**Fig. 7:**
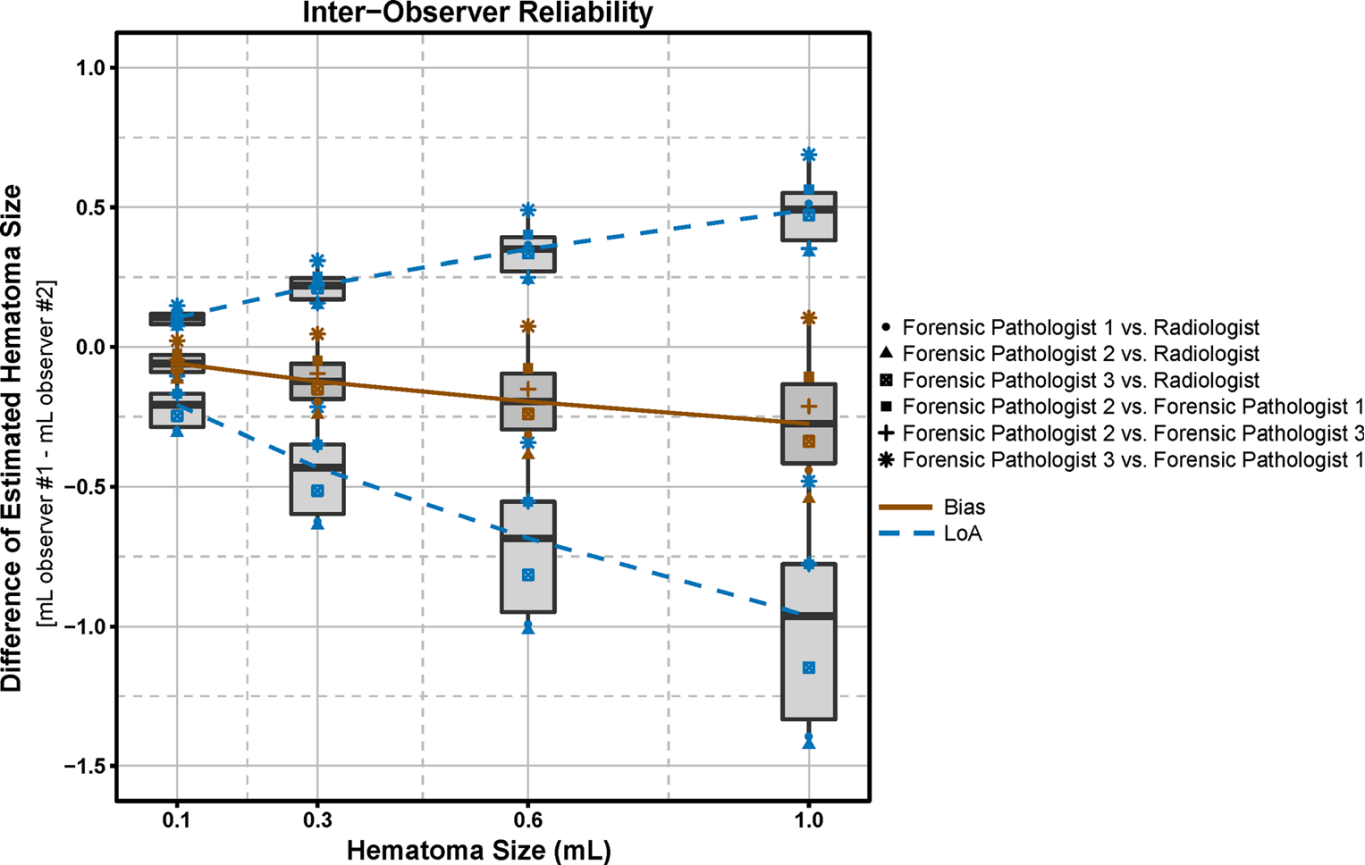
Bland-Altman plot describing inter-observer reliability of summarized results of transformed data (hematomas located in the subcutaneous fatty tissue). The *brown symbols* represent the averaged bias over the estimated hematoma volumes of two observers. The *blue symbols* represent the LoA (Limits of Agreement; bias ±1.96 × standard deviation) of the estimated hematoma volumes of 2 observers. The *boxes* represent the median, 25th and 75th percentiles of the averaged data (bias and LoA) over all calculations. The *brown curve* links the bias averaged over all observers (*thick black line* in boxes), the *blue dashed curves* link the averaged LoA of all observers

The results for the assessment of inter-observer reliability are presented in Fig. 7. The median LoA ranged from −0.21 to 0.11 mL for 0.1 mL hematomas and −0.96 to 0.49 mL for 1.0 mL. There was a small negative bias in all sequences, with TIR and T1T2w TSE showing the lowest LoA (Online Resource 3). Figure 7 depicts that the agreement between the forensic pathologists is generally better than between forensic pathologists and the radiologist.

## Discussion

Blunt force injuries, such as hematomas, are a matter of special interest in forensic medicine. Although hematomas do not usually have any therapeutic consequence, their presence can have relevant implications for forensic reconstruction and verification of a specific course of events.

In order to assess the detection limit of blood volumes in subcutaneous tissue, artificial hematomas were experimentally evaluated using different MR sequences in a porcine tissue model. Firstly, the segmentability of the four studied MR sequences was evaluated, followed by examination of accuracy and precision of the volume estimation. When comparing the four sequences regarding detection of hematomas, the present study demonstrated that the TIR sequence exhibited the best segmentability rate. The most accurate results regarding volume estimation could be achieved with the T1T2w TSE sequence. In intra- and inter-observer reliability experiments the TIR and T1T2w TSE sequences showed the most reproducible results.

In general, with all four MR sequences suitable contrast properties for segmenting hematomas in the subcutaneous fatty tissue was achieved. Thus, the applicability of the selected sequences for image segmentation and volume measurement was shown. A crucial requirement for the choice of the sequences was to achieve good contrast between the lesion and the fatty tissue. The contrast achievable between different tissues is influenced by several factors, with the main factors being the relaxation behavior of each tissue. Relaxation times (T1 and T2) are intrinsic tissue properties which describe the complex process of nuclear spin magnetization returning to its equilibrium state following excitation in MRI. Additional factors relevant in achieving contrast are for example the magnetic properties (e.g. susceptibility) of tissues and saturation of specific tissue types (e.g. fat), which is dependent on the applied MR sequence.

In the gradient echo sequence (FLASH) high contrast was observed due to the differing magnetic properties of deoxygenated compared to oxygenated hemoglobin. These intrinsic tissue properties led to local magnetic field inhomogeneities, which in turn led to signal loss during the application of this imaging sequence. The applied turbo inversion recovery sequence (TIR) allowed nulling of the fat signal at an inversion time (TI) of 200 ms (3.0 T) based on the characteristically short T1 of fat. Due to the longer T1 relaxation time of venous blood, which is a major component in hematomas, the signal contribution of the lesion was not nulled enabling acquisition of images with good contrast between the hematoma and the suppressed fatty tissue. In the proton-density weighted sequence with fat saturation (PDw TSE FS) a special pulse was used to abrogate the fat signal, so that contrast between the lesion and the fatty tissue was once again maximized. In the sequence with combined T1 and T2 weighting (T1T2w TSE) the fatty tissue appeared bright due to its short T1 and rather long T2 relaxation time. In comparison, deoxygenated blood, present in hematomas, appeared darker due to its longer T1 and shorter T2 relaxation times, once again leading to good contrast in this sequence.

The contrast between the hematoma located in the muscle tissue and the actual muscle tissue itself was not adequate. This was reflected in the results of the segmentability of all lesions (intramuscular and subcutaneous fatty tissue), which demonstrated a generally lower segmentation rate. The only sequence having no location dependency was TIR, where all hematomas could be segmented independently of their location. This can be explained by the differences of the T1 relaxation times of the corresponding tissues (fatty tissue: 400 ms, muscle 900 ms, venous blood: 1500–1650 ms) [35, 36]. The other sequences did not allow a 100% segmentation rate of all hematomas but did show increased segmentability, when only hematomas in fatty tissue were taken into account.

The overall accuracy and precision of the volume estimation was poor. Averaged volumes were generally overestimated, if compared to the injected volume. If evaluating the lesions inside the fatty tissue only, there was an increase in accuracy and precision, probably due to the improved contrast between subcutaneous fatty tissue and blood. However, the volumes of lesions in the subcutaneous fatty tissue were still overestimated by all observers, especially by the radiologist. Over- and underestimation is a general issue in volume determination using imaging techniques in a medical context. Huttner et al. [37] described significant overestimation of irregularly shaped cerebral hematomas in CT images using ABC/2 technique for volume estimation. A recent study by Leddy et al. [38] noted a significant overestimation of MRI in volume estimation of breast cancer in contrast to other imaging modalities. In volume estimation of prostate cancer using different MR sequences, over- and underestimation of the actual tumor volume was observed depending on the MR sequences used, with no single sequence resulting in accurate tumor volume determination [39]. The volume overestimation observed in the present study can be explained by several factors. The inner part of the hematoma (as seen in Fig. 1) is a compact volume mainly consisting of blood, whereas in the periphery the blood intermingles with fatty tissue, dispersing around the fat lobuli and along the septa. The appearance of this outer area in MR images is dependent on the contrast and windowing settings. The chosen window parameters have a great impact on the segmentation results and therefore fixed window values were introduced in this study. Nevertheless, the individual perception of hematoma perimeters in MR images remains a main source of error in manual segmentation. Another reason for the overestimation of hematoma volumes might originate from partial volume effects, as a consequence of the limited voxel size. Especially manual segmentation by the radiologist in contrast to non-radiologist segmentation showed extended perimeters of the hematoma. Interestingly, the sequence where the least hematomas were segmented (T1T2w TSE) was the most accurate one concerning volume estimation. In the T1T2w TSE sequence fatty tissue structures were visible within some hematomas, which were not visible in the other sequences (Fig. 3), additionally leading to a better defined differentiation of the hematomas. Another effect on volume estimation could also be attributed to image resolution, which was higher for the T1T2w TSE and PDw TSE FS sequences than for the FLASH and TIR sequences. However, this effect was more prominent for the T1T2w TSE sequence.

A decreasing relative error for all observers was observed with increasing hematoma size. This can be explained by the physical model used to normalize the data. By assuming a hematoma of spherical form, the resulting error due to *over-segmentation* decreases with increasing hematoma radius. In this study, very small amounts of blood were examined. However, under in vivo conditions usually larger hematoma volumes would be expected, which would reduce this relative error. These factors which contributed to the volume overestimation can also be held accountable for the low intra- and inter-observer reliability obtained in this study.

One limitation of this study was that for some hematomas residual blood flowed out of the injection canal or was not completely injected leading to minor errors in the ground truth. Additionally, the acquisition protocols were optimized for achieving good contrast between blood and fatty tissue. Therefore, blood in muscle tissue was not optimally depicted.

In conclusion, this study demonstrates the potential of MRI to visualize even very small amounts of blood in subcutaneous fatty tissue. Hematomas were in general well segmentable in all of the applied sequences, with the TIR sequence showing the best segmentation rate independent of the location of the hematomas. Concerning volume estimation, the T1T2w TSE sequence was most accurate. Overall, the TIR and T1T2w TSE sequences were identified as the sequences with the highest potential for hematoma detection and volume estimation – even for very small hemorrhages.

In future, these results can be used to improve MR protocols for hematoma detection in both, forensically and clinically relevant cases. Therefore, this study provides the first step to optimize clinical forensic imaging using MRI, especially in cases where objective evidence of subcutaneous hematomas is required.

## Key points

1. Reliable characterization of hematomas in the subcutaneous fatty tissue is necessary for the forensic reconstruction in cases of interpersonal violence or child maltreatment.
2. Four magnetic resonance sequences (FLASH, TIR, PDw TSE FS, T1T2w TSE) were evaluated with respect to the contrast between hematoma and subcutaneous fatty tissue and volume estimation of artificial hematomas in a porcine tissue model.
3. The TIR sequence exhibited the best segmentability rate and the T1T2w TSE sequence showed the most accurate results regarding volume estimation.
4. Segmentation and volume estimation of artificial hematomas of various sizes (0.1–1 mL) were successfully achieved using MRI.

## Electronic supplementary material

ESM 1 (PDF 117 kb)

ESM 2 (PDF 300 kb)

ESM 3 (PDF 340 kb)
