# Supplementary material for: Detection and volume estimation of artificial hematomas in the subcutaneous fatty tissue: comparison of different MR sequences at 3.0 T
Source: Forensic Sci Med Pathol. 2017 Mar 1;13(2):135–44. doi: 10.1007/s12024-017-9847-8 (PMC5429378; doi:10.1007/s12024-017-9847-8)
Supplement: Supplementary file 1 — (PDF 117 kb) [file 12024_2017_9847_MOESM1_ESM.pdf]

Online Resource 1 Graphic representation of the physically motivated normalization used for transformation of the gained data for statistical analysis

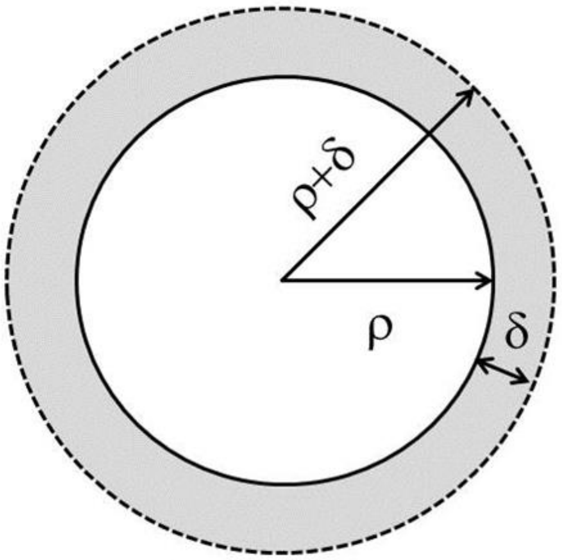

$$Error = \frac{4\pi}{3}(\delta^3 + 3\delta^2\rho + 3\delta\rho^2)$$

$$Error \propto 3\delta\rho^2 \propto \sqrt[3]{V^2}$$
