# Supplementary material for: Detection and volume estimation of artificial hematomas in the subcutaneous fatty tissue: comparison of different MR sequences at 3.0 T
Source: Forensic Sci Med Pathol. 2017 Mar 1;13(2):135–44. doi: 10.1007/s12024-017-9847-8 (PMC5429378; doi:10.1007/s12024-017-9847-8)

Online Resource 2 Bland-Altman plots describing intra-observer reliability of transformed data (hematomas located in the subcutaneous fatty tissue) for each MR (Magnetic Resonance) sequence separately. Brown symbols represent the averaged bias over the estimated hematoma volumes of each observer, out of 2 measurements. The blue symbols represent the LoA (Limits of Agreement; bias ± 1.96 × standard deviation) of the estimated hematoma volumes of each observer. The boxes represent the median, 25% and 75% quartiles of the averaged data (bias and LoA) over all observers. The brown continuous curve links the bias averaged over all 4 observers (thick black line in boxes), the blue dashed curves link the averaged LoA of all observers

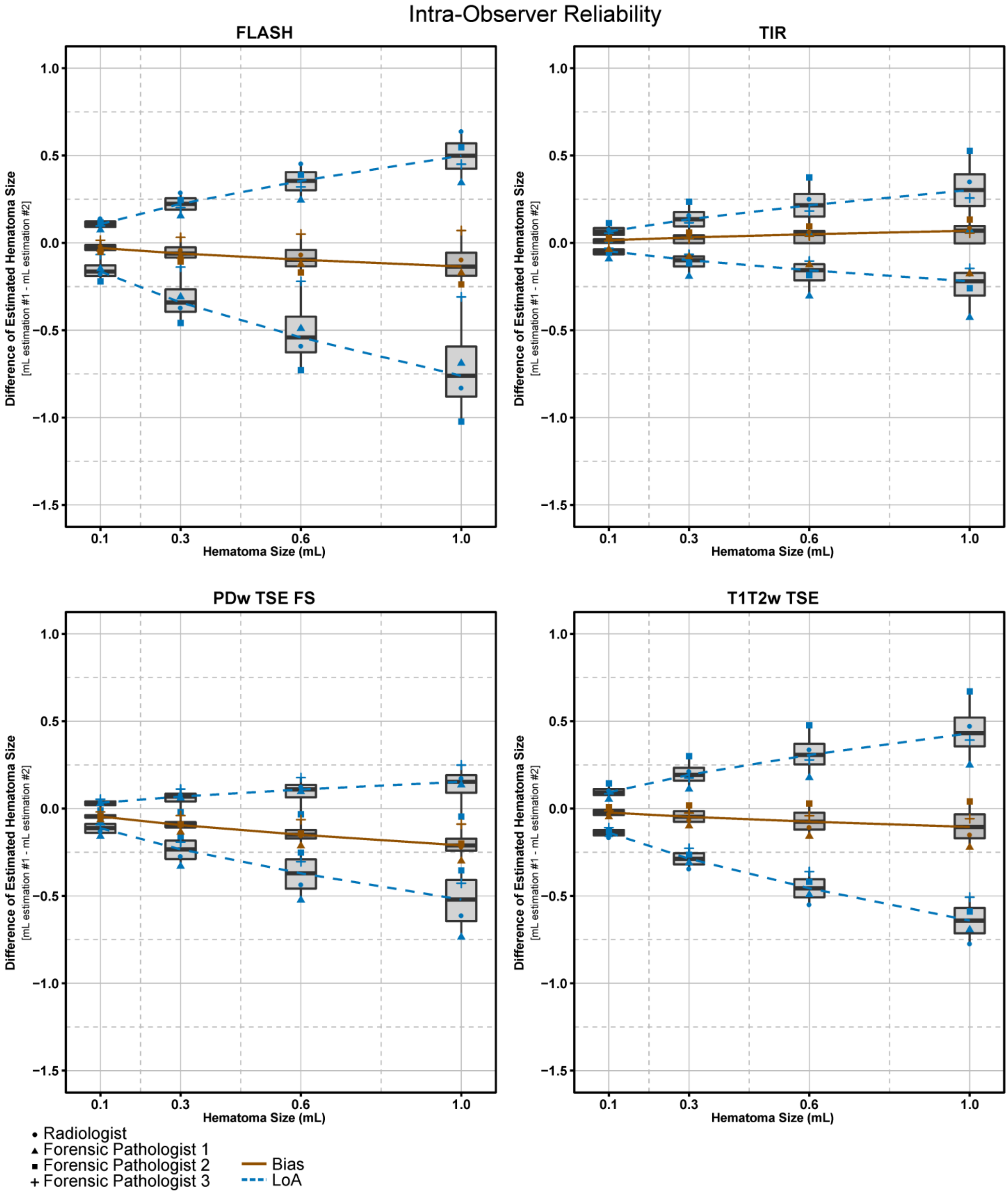

Supplement: Supplementary file 2 — (PDF 300 kb) [file 12024_2017_9847_MOESM2_ESM.pdf]
