# Supplementary material for: Detection and volume estimation of artificial hematomas in the subcutaneous fatty tissue: comparison of different MR sequences at 3.0 T
Source: Forensic Sci Med Pathol. 2017 Mar 1;13(2):135–44. doi: 10.1007/s12024-017-9847-8 (PMC5429378; doi:10.1007/s12024-017-9847-8)

Online Resource 3 Bland-Altman plots describing inter-observer reliability of transformed data (hematomas located in the subcutaneous fatty tissue) for each MR sequence separately. The brown symbols represent the averaged bias over the estimated hematoma volumes of two observers. The blue symbols represent the LoA (Limits of Agreement; bias ± 1.96 × standard deviation) of the estimated hematoma volumes of 2 observers. The boxes represent the median, 25% and 75% quartiles of the averaged data (bias and LoA) over all calculations. The brown curve links the bias averaged over all observers (thick black line in boxes), the blue dashed curves link the averaged LoA of all observers

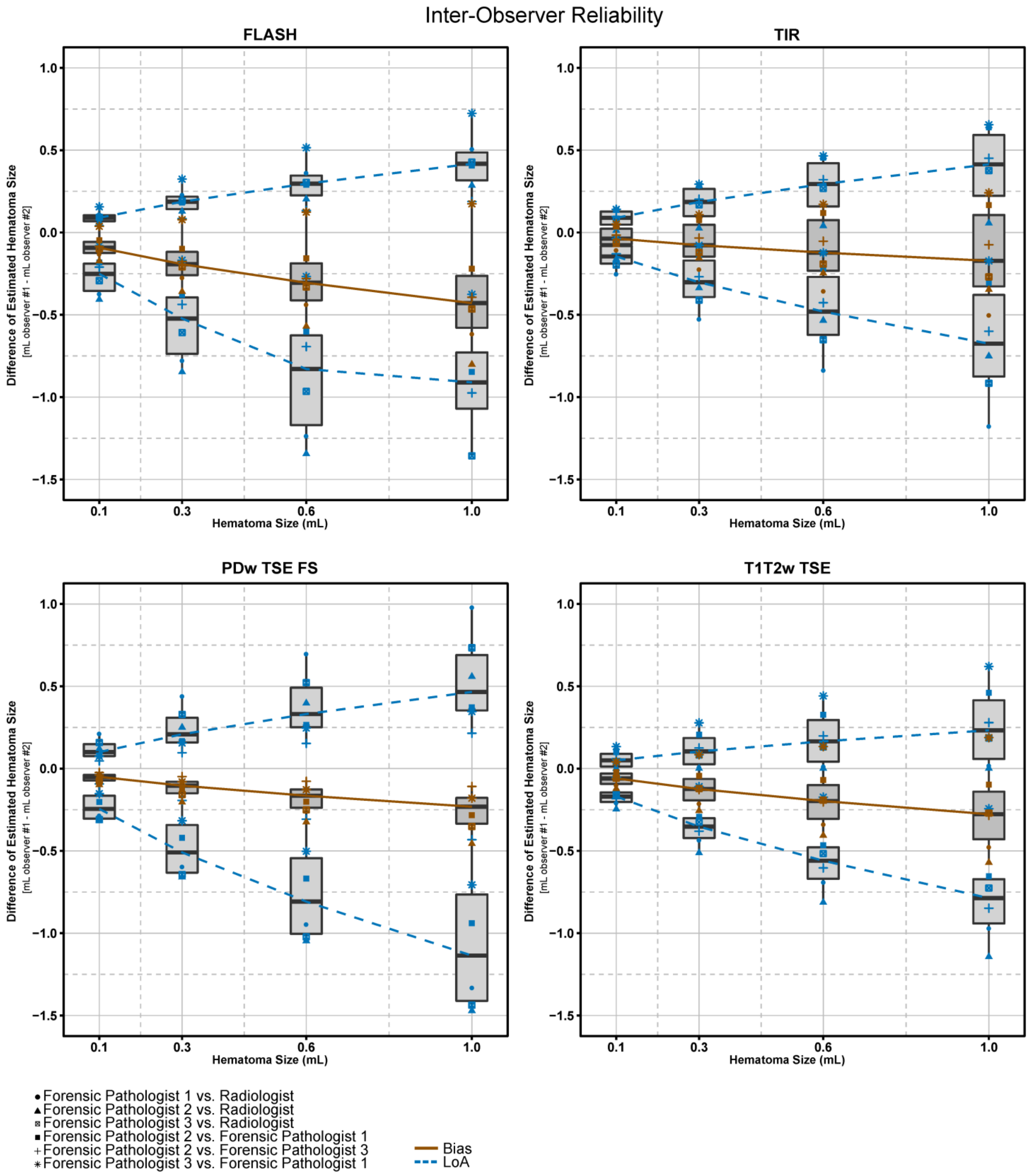

Supplement: Supplementary file 3 — (PDF 340 kb) [file 12024_2017_9847_MOESM3_ESM.pdf]
